# Attenuated Total Reflectance Fourier Transform Infrared Spectroscopy (FTIR) and Artificial Neural Networks Applied to Investigate Quantitative Changes of Selected Soluble Biomarkers, Correlated with *H. pylori* Infection in Children and Presumable Consequent Delayed Growth

**DOI:** 10.3390/jcm9123852

**Published:** 2020-11-27

**Authors:** Weronika Gonciarz, Łukasz Lechowicz, Mariusz Urbaniak, Wiesław Kaca, Magdalena Chmiela

**Affiliations:** 1 Department of Immunology and Infectious Biology, Institute of Microbiology, Biotechnology and Immunology, Faculty of Biology and Environmental Protection, University of Lodz, Banacha 12/16, 90-237 Łódź, Poland; weronika.gonciarz@biol.uni.lodz.pl; 2 Institute of Biology, Faculty of Natural Sciences, Jan Kochanowski University, Uniwersytecka 7, 25-406 Kielce, Poland; lukasz.lechowicz@ujk.edu.pl (Ł.L.); wieslaw.kaca@ujk.edu.pl (W.K.); 3 Institute of Chemistry, Faculty of Natural Sciences, Jan Kochanowski University, Uniwersytecka 7, 25-406 Kielce, Poland; mariusz.urbaniak@ujk.edu.pl

**Keywords:** *H. pylori*, FTIR, children

## Abstract

*Helicobacter pylori* infections causing gastroduodenal disorders are a common medical problem. The aim of this study was to determine the specific motives of infrared spectroscopy (IR) spectra of sera from *H. pylori*-infected and uninfected children applied to investigate quantitatively-selected soluble biomarkers correlated with *H. pylori* infection in children and presumable consequent delayed growth. Sera from 41 children infected with *H. pylori* (Hp(+)) and 43 uninfected (Hp(−)) under the care of the Polish Mother’s Hospital in Lodz, Poland, were analyzed. The *H. pylori* status was confirmed by gastroscopy, ^13^C urea breath testing, and anti-*H. pylori* IgG antibodies. Infrared spectra were measured using an FTIR/FT-NIR Spectrum 400 spectrometer (PerkinElmer). The IR spectrum was measured in the wavenumber range 3000–750 cm^−1^ and subjected to mathematical calculation of the first derivative. Based on the chi-square test, 10 wavenumbers of spectra correlating with *H. pylori* infection were selected for use in designing an artificial neural network. Ten parts of the IR spectra correlating with *H. pylori* infection were identified in the W2 and W3 windows associated mainly with proteins and the W4 window related to nucleic acids and hydrocarbons. Artificial neural networks for *H. pylori* infection were developed based on chemometric data. By mathematical modeling, children were classified towards *H. pylori* infection in conjunction with elevated levels of selected biomarkers in serum potentially related to growth retardation. The study concludes that IR spectroscopy and artificial neural networks may help to confirm *H. pylori*-driven growth disorders in children.

## 1. Introduction

*Helicobacter pylori* is a Gram-negative pathogenic bacterium that specifically colonizes gastric mucosa in humans (average frequency of infection 50%) and was described by Warren and Marshall in 1983 [1]. These bacteria if not eradicated can persist for life. In the stomach, *H. pylori* induce excessive inflammatory response, leading to different disorders such as: gastric and duodenal ulcers, chronic gastritis, and malignant diseases including MALT (mucosa-associated lymphoid tissue) lymphoma, and gastric cancer [2,3,4,5,6]. *H. pylori* induces gastritis in all infected individuals; however, clinical symptoms occur in only 10–15% of cases. The course of infection depends on the virulence factors of *H. pylori*, the susceptibility of the individual host, and socio-economic status [2,4]. *H. pylori* infections are chronic, which indicates that the host immune mechanisms, both humoral and cellular, are not effective in combating these infections. Long-term infections—especially with *H. pylori* CagA+ strains producing CagA (cytotoxin associated gene A) protein—in conjunction with excessive local inflammatory response in the gastric tissue may also contribute to the development of systemic inflammation and extragastric diseases such as immune thrombocytopenic purpura, iron deficiency anemia, and vitamin B12 deficiency [7,8,9,10]. Other diseases such as cardiovascular disorders, diabetes mellitus, dermatological diseases, neurologic disorders, and even lung cancer are thought to be linked with *H. pylori* infection [11,12,13,14,15]. The relation between *H. pylori* infections and growth retardation in children has been suggested to be due to iron deficiency or antigenic mimicry between *H. pylori* compounds and appetite-regulating peptides, thrombocyte proteins, or due to modulation of ghrelin and leptin secretion [16,17,18,19,20]. In children, symptoms of gastritis may include nausea, vomiting, and abdominal pain. Children suffering from peptic ulcer disease can have ulcers that bleed, causing hematemesis (bloody vomit) or melena (bloody stool). Younger children with peptic ulcers may not have such clear symptoms, so their illness may be difficult to diagnose. The European Consensus Group (ECG) during a meeting in Maastricht in 2002 recommended the urea breath testing (UTB) ^13^C and histological examination of gastric tissue specimens as major diagnostic methods [21]. Testing of stool samples for *H. pylori* antigens was also recommended, particularly in fully symptomatic patients [22]. Although these methods are sensitive and specific enough to detect infection, they cannot investigate the systemic changes in the level of soluble components correlated with *H. pylori* infection in children as a presumable cause of delayed growth. Growth failure can occur for various reasons. Finding metabolic markers that change during *H. pylori* infection may help to confirm the infectious background of delayed growth in children. Exposure to *H. pylori* infection may upregulate various biocomponents, both locally and systemically. Fourier transform infrared spectroscopy (FTIR) is a fast physical technique that can be used for the qualitative and quantitative analysis of biological fluids like blood, serum, saliva, and urine, and for monitoring cellular alterations [23,24,25,26]. The FTIR spectrum of biological samples such as human serum can be divided into groups of components with typical absorption bands in the wavenumber windows (W): W1—fatty acids (wavenumber range 3000–2800 cm^−1^), W2—peptides and proteins (wavenumber range 1800–1500 cm^−1^), W3—proteins, phosphate-carrying compounds, and fatty acids (wavenumber range 1500–1200 cm^−1^), W4—carbohydrates (wavenumber range 1200–900 cm^−1^). The W5 absorption band (wavenumber range 900–750 cm^−1^) corresponds to specific peaks unique to the sample [27,28,29,30]. The aim of this study was to determine the specific motives of IR spectra for children’s sera from *H. pylori*-infected vs. uninfected subjects, which may help to investigate molecular-level changes correlated with this infection in children and presumable consequent delayed growth.

**Figure 1:**
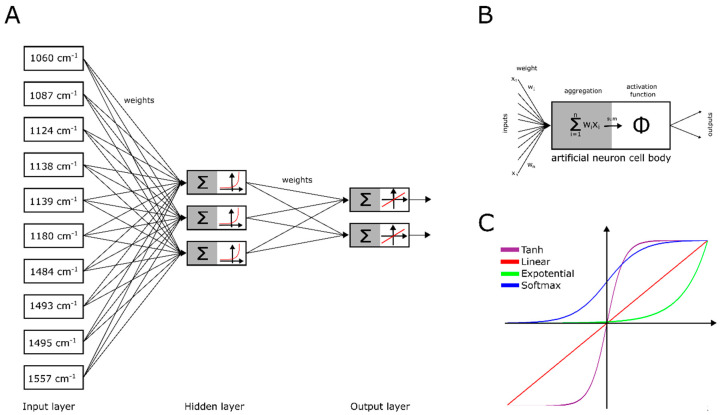
Artificial neural network topology based on the best network for detecting *Helicobacter pylori* infection. (**A**) Topology of an artificial neural network. (**B**) Detailed structure of an artificial neuron. (**C**) The most common activation functions of an artificial neuron.

Artificial neural networks (ANNs) are mathematical models inspired by the structure of the nervous system. Artificial neural networks have been applied for chemometric data analyses (e.g., in testing the sensitivity of bacteria to antibiotics or identifying infectious agents) [31,32,33,34,35]. Artificial neural networks are made of many artificial neurons arranged in several layers: an input layer, a hidden layer(s) (usually one to three), and an output layer (Figure 1A). The main component of artificial neural networks is the artificial neuron (Figure 1B). The structure of an artificial neuron includes several inputs (x_1_–x_n_) corresponding to dendrites in a biological cell and anywhere from one to several outputs. Input data (e.g., absorbance of the IR spectrum) is modified by synaptic weights (w_1_–w_n_) and then transferred to an artificial neuron (“cell body”). The artificial neuron consists of two elements: an aggregator that sums up signals and an activator that modifies the signal by using mathematical functions (Figure 1C). The artificial neural network learning process is based on presenting the ANN with the data included in the training set, and then calculating the errors made by the network. Then, the synaptic weights are corrected and the whole process starts all over again. After reaching the required level of the number of correct classifications, the sensitivity and specificity of the ANN is verified by presenting its cases from the validation set [36].

## 2. Material and Methods

### 2.1. Patients and Controls

Approval for the study was obtained from the Bioethical Committee in the Polish Mother’s Memorial Hospital—Research Institute (PMMH-RI) in Lodz (RNN/134/13/KE/2-13). Healthy children—the control group (43 individuals of both sexes, mean age 11.5 ± 2.8)—and children with *H. pylori*-related gastritis (41 individuals of both sexes, mean age 12.5 ± 3.3) were under the care of the PMMH-RI in Lodz. Informed consent was obtained for experimentation. The right to privacy of participants was guaranteed. Blood samples were collected in a fasting state, on admission, before medical and pharmacological intervention. Serum samples were obtained within 1 h, by 30 min incubation at room temperature and subsequent 30 min incubation at 4 °C followed by centrifugation (2000× *g*, 10 min, 4 °C). The sera were aliquoted and stored at −80 °C for further analysis. Samples were thawed directly before being used in experiments.

### 2.2. Diagnosis of H. pylori Infection

The *H. pylori* status in patients and healthy donors was estimated by the ^13^C urea breath test (^13^C UBT) [37], immunoblotting for anti-*H. pylori* IgG (Milenia^®^Blot *H. pylori*, DPC Biermann, GmbH, Bad Nanheim, Germany), and the enzyme-linked immunosorbent assay (ELISA) for IgG antibodies against the *H. pylori* antigenic complex glycine extract (GE), which was obtained by extraction with a glycine acid buffer of surface antigens from the reference *H. pylori* strain CCUG (Culture Collection University of Gothenburg, Sweden) 17,874, producing cytotoxin associated gene A (CagA) protein and vacuolating cytotoxin (VacA), as previously described [38]. Major proteins in GE recognized by the reference sera from *H. pylori*-infected patients were as follows: 120 kDa (CagA), 87 kDa (VacA), 66 kDa (UreB), 60 kDa (Hsp), 29 kDa (UreA), between 66–22 kDa [39]. The GE protein concentration was 600 μg/mL (NanoDrop 2000c Spectrophotometer, ThermoScientific, Waltman, WY, USA) and <0.001 EU/mL of LPS, as shown by the chromogenic Limulus amebocyte lysate test (Lonza, Braine-Alleud, Belgium). The serum samples were also examined for the presence of anti-CagA IgG using recombinant CagA (rCagA, courtesy of Dr. Antonello Covacci, IRIS, Siena, Italy) as previously described [39]. Furthermore, ^13^C UBT-positive children with dyspeptic symptoms underwent gastroscopy and routine histological examination of gastric tissue specimens towards *Helicobacter*-like organisms and inflammatory response. Short status in children was measured according to population standards [40]. A height standard deviation score (H SSD) of over −2.0 qualified children for the short status group. In the group of uninfected children there were no children with short stature. By comparison, in the group of *H. pylori*-infected children 36 showed a growth retardation.

### 2.3. The Measurement of Infrared Spectra and Their Processing

IR spectra of human sera were measured by using the attenuated total reflectance (ATR) spectroscopy technique. Measurements were performed at 20 °C at constant air humidity. Serum samples were thawed at room temperature and then shaken for 30 s. One microliter of serum was added to the spectroscope crystal and left for 5 min to evaporation. The IR spectra were measured in the range of wavenumbers 4000–650 cm^−1^ with a resolution of 1 cm^−1^ and then pre-processed in two steps: (a) calculation of the first derivative by five-point stencil, (b) normalization to the range {0, 1} [41].

### 2.4. Mathematical Model Development for Patient Differentiation

The chi-square statistical test was used to check the part of the IR spectra which correlated with the examined feature. Next, a number of mathematical models based on multilayer perceptrons (a type of artificial neural network (ANN)) were built. The individual artificial neural networks differed from each other in complexity—the number of input neurons (from 1 to 10) and hidden neurons (from 1 to 5), the activation functions of neurons, and the function of error. Two output neurons were selected for analysis; these neurons corresponded to two opposite states concerning *H. pylori* infection: Infected or uninfected. The input data for the ANNs were the absorbance values of the wavenumbers, which strongly correlated with the examined feature. The total set of spectra was randomly divided into two separate subsets: Learning and validating. The quality of the network was evaluated on the basis of the number of correct classifications in the validation set. The calculations were carried out using Statistica 12 software (StatSoft, Round Rock, TX, USA). Selected ANNs were validated in terms of sensitivity, miss rate, specificity, false positive rate, precision, false discovery rate, false omission rate, negative predictive value, positive likelihood ratio, negative likelihood ratio, accuracy, informedness, and markedness.

### 2.5. Statistical Analysis

Results are expressed as the median values with a range. The differences between tested variables were assessed using Statistica 12 software with a nonparametric Mann–Whitney U test. The results were considered statistically significant when *p* < 0.05.

## 3. Results

### 3.1. H. pylori Serological Status in Children

**Figure 2:**
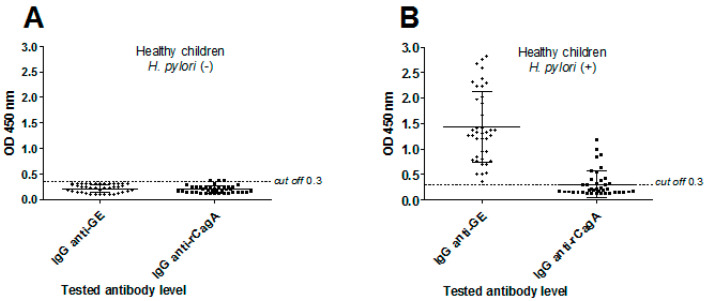
The prevalence and levels of anti-*H. pylori* antigenic complex glycine extract (GE) and anti-CagA, IgG antibody production in *H. pylori*-uninfected or *H. pylori*-infected children. (**A**) Healthy children seronegative for anti-*H. pylori* antibodies—*H. pylori* (−), *n* = 43; (**B**) Children infected with *H. pylori*, seropositive for anti-*H. pylori* antibodies—*H. pylori* (+), *n* = 41. Shown are mean values ± standard deviation (SD).

Differences between study groups in IR spectroscopy were related to *H. pylori* infection and anti-*H. pylori* IgG. The panels of sera from healthy children (43 individuals) and from children with *H. pylori*-induced gastritis confirmed by histological examination of gastric tissue specimens (41 individuals) were screened (Figure 2). The serum samples from healthy children did not contain anti-GE IgG and were free from anti-CagA IgG (Figure 2A). By comparison, 40 sera of children with diagnosed gastritis due to *H. pylori* infection were positive for anti-GE IgG and among them 14 sera contained anti-CagA IgG (Figure 2B).

### 3.2. Analysis of IR Spectra of Human Sera

**Figure 3:**
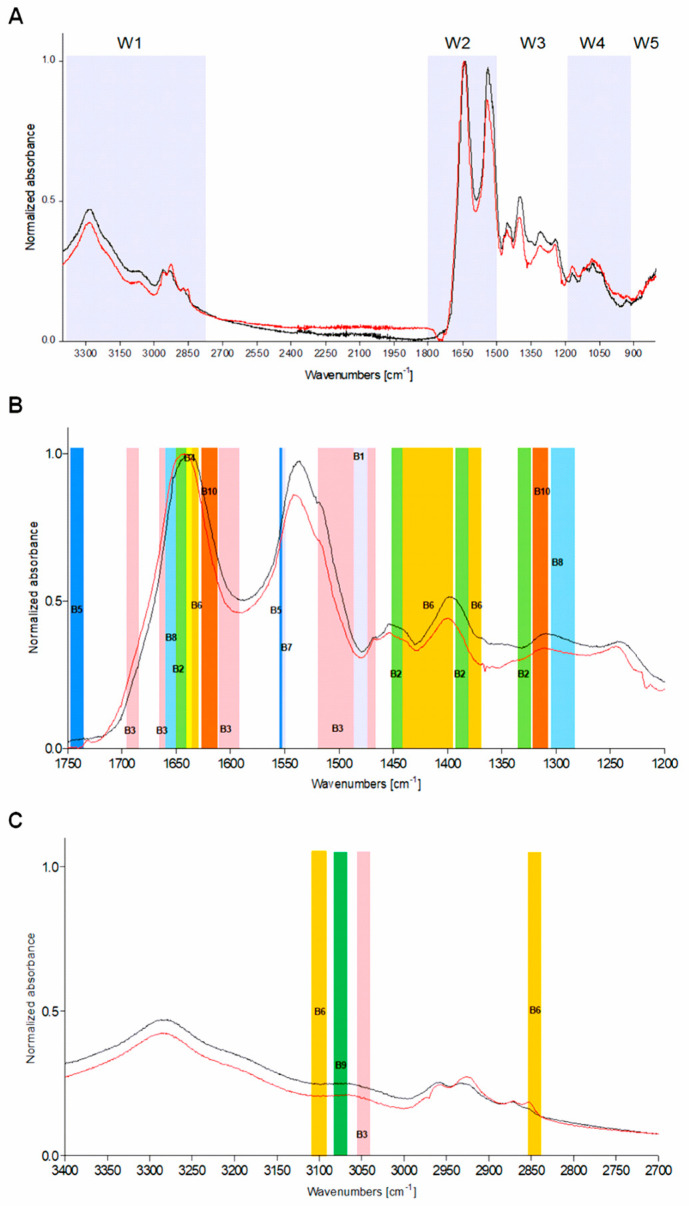
Representative infrared spectra of children sera: red indicates *H. pylori*-positive and black indicates *H. pylori*-negative individuals. (**A**) Representative total spectra. Windows W1–W4 correspond to the characteristic components: amino acids and fatty acids; peptides and proteins; proteins, phosphate-carrying compounds and fatty acids; and carbohydrates, respectively, while W5 corresponds to sample-specific peaks. (**B**,**C**) Parts of IR spectra differentiating *H. pylori*-positive vs. *H. pylori*-negative subjects. B1–B15—characteristic absorption bands differentiating the study groups.

The IR spectra of serum samples can be divided into groups of components with characteristic absorption bands: fatty acids (window W1: the wavenumber range 3000–2800 cm^−1^); peptides and proteins (W2: 1800–1500 cm^−1^); proteins, phosphate-carrying compounds, and fatty acids (W3: 1500–1200 cm^−1^); and carbohydrates (W4: 1200–900 cm^−1^). The W5 part (wavenumber range 900–750 cm^−1^) corresponds to specific peaks unique to the sample (Figure 3).

Vibration band assignment was carried out on the infrared spectra of sera by comparing the position, relative intensity, and shape of the bands with the corresponding features of bands of related molecules. Infrared spectra of serum samples from healthy children vs. children infected with *H. pylori* are shown in Figure 3. Absorption bands for proteins, lipids, and carbohydrates were taken into account during analysis. Particularly specific molecules, which were identified in the composition of IR spectra of sera, such as: α-tocopherol (window B1), β-carotene (window B2), folic acid (window B3), ghrelin (window B4), leptin (window B5), lycopene (window B6), lutein (window B7), vitamin B6 (window B8), vitamin B12 (window B9), and vitamin C (window B10) allowed the differentiation between *H. pylori*-infected and uninfected individuals (Figure 3A–C, Table 1).

The ID column shows representative characteristic parts of infrared spectra of children’s sera and molecules differentiating *H. pylori*-infected vs. *H. pylori*-uninfected children.

A vibration band assignment was done to analyze the frequencies of chemical groups present in the sample. As shown in Table 1, in the spectral region (B1) corresponding to α-tocopherol the prominent absorption peak 1472 cm^−1^ is due to the CH_3_/CH_2_ asymmetrical scissoring (peptides and proteins), while 1078 cm^−1^ corresponds to C-O symmetric stretching of glucose region (carbohydrates). The absorption band B2 (component group: amide I and amino acid) is characteristic for β-carotene absorption vibration peaks: 1650 cm^−1^ C=O symmetric stretching, 1457 cm^−1^ asymmetric C-H scissoring of CH_3_, 1384 cm^−1^ CH_3_ deformation, 1324 cm^−1^ O-H bending, and 1096 cm^−1^ C-C-C bending vibrations (Figure 3, Table 1). The spectral region B3, which is typical for peptides and proteins (component group amide I, amino acid), corresponds to a vibration band characteristic for folic acid. Typical parameters of this region are as follows: N-H (amino acid) and CO_2_ asymmetric stretching (3050 cm^−1^, 1485–1519 cm^−1^), NH_2_ (amide I) scissoring (1604–1619 cm^−1^, 1650 cm^−1^), and C=O symmetric stretching (1693 cm^−1^) vibrations. The prominent absorption peak identified for ghrelin was 1640 cm^−1^ (B4) due to the NH_2_ scissoring (amide I) of proteins whereas the absorption peaks for leptin were identified at wavenumbers 1740 cm^−1^ and 1550 cm^−1^ (B5). The C=O stretching and N-H bending vibration strongly coupled to C-N stretching vibration of this protein. The absorption bands for lycopene (B6) at 3100 cm^−1^ correspond to N-H asymmetric stretching (amino acid), whereas wavenumber 2851 cm^−1^ is attributed to lipids C-H symmetric stretching of CH_2_ group, 1640 cm^−1^ corresponds to NH_2_ scissoring, 1450–1400 cm^−1^ to CH_3_ asymmetric deformation, and 1375 cm^−1^ to CH_2_ wagging vibrations of component group amide I. The 1517–1500 cm^−1^ (B7) is predominantly linked to CO_2_ asymmetric stretching vibrations of peptides and proteins, and characteristic for lutein. The spectral region 1280–1315 cm^−1^ (B8, vitamin B6) is predominantly linked to C-H/N-H deformation vibration models methyl groups, vibrations of phospholipids of proteins, phosphate-carrying compounds, and fatty acids (Figure 3A,B, Table 1). The asymmetric and symmetric stretching CH_3_ asymmetric stretching of CH_3_ group and C=O band vibrations were found at 3120 cm^−1^ and 1670–1665 cm^−1^ (B9, Vitamin B12). The characteristic vibration bends were found for the vitamin C (B10): 1760 cm^−1^ C=O stretching, 1634 cm^−1^ NH_2_ scissoring (amide I), and 1322 cm^−1^ C-H/N-H deformation vibration models methyl groups (amide III) (Figure 3A–C, Table 1).

### 3.3. Wavenumbers Correlating with H. pylori Infection and Mathematical Models Identifying Sera of Infected Individuals

By using the chi-square statistical test, it was possible to extract parts of IR spectra correlating with *H. pylori* infection in children. For designing artificial neural networks, 10 wavenumbers which strongly correlated with this infection were considered (Table 2). Six wavenumbers were presented in the W4 window, which were associated with vibrations derived from hydrocarbons and nucleic acids. Three wavenumbers correlated with *H. pylori* infection in the W3 window. This window is associated with different chemical bonds (the so-called mixed region); however, proteins and phosphorus-containing compounds are key for this window. The wavenumber 1557 cm^−1^, localized within the W2 window, was strongly associated with *H. pylori* infection. According to the literature, the W2 window illustrates proteins and peptides. In *H. pylori*-infected individuals it may correspond to antibody production and/or an increased production of inflammatory proteins.

Satisfactory results of IR spectra analysis were obtained using ANNs. Proposed models are characterized by 83–90% accuracy. The topology of the best ANNs is presented in Table 3. However, by increasing the size of the studied groups, the differentiation capacity of the ANNs could be increased.

The best neural network contained 10 entry neurons, 3 hidden neurons, and 2 output neurons (10-3-2 topology). The quality of the perceptron was assessed on the basis of 13 determinants included in Table 4. This network reached 95% sensitivity and 86% specificity, indicating the certain potential of chemometric methods in differentiating *H. pylori*-infected from *H. pylori*-uninfected children on the basis of IR spectrum analysis.

## 4. Discussion

IR spectroscopy has become a suitable method for the characterization of biological materials, like tissue sections, cytological and histological specimens, or biofluids. Liu et al. identified leukemia cells CLL through the level of DNA/RNA and lipids [51]. Zhou et al. showed spectral changes in the HL60 cell line during differentiation and apoptosis [52]. Erukhimovitch et al. applied FTIR to the analysis of human plasma samples in order to detect spectral parameters that might serve as biomarkers for the monitoring and identification of patients with leukemia [53]. Shen et al. developed a noninvasive method for the measurement of glucose concentration in whole blood samples of diabetes patients [54]. Sankari et al. analyzed serum immunoglobulins IgA, IgM, and IgG in patients with myeloma using FTIR [55], whereas Lechowicz et al. used FTIR spectroscopy to differentiate rheumatoid arthritis (RA) from non-RA sera [41]. FTIR can potentially be useful for monitoring chronic infections and related consequences on the basis of systemic changes in concentration of exo- and endogenous molecules.

*H. pylori* induces gastritis, gastric or duodenal ulcers, and even gastric cancers in infected individuals. About 20% of infected people develop clinical symptoms. The reason for such different responses to *H. pylori* is not well understood. In children *H. pylori* infection can cause developmental disorders. It can affect the balance of nutritional status in children and correlates with malnutrition and growth retardation [20,56,57,58,59]. Symptomatic infections are correlated with deficiencies of macro- and micronutrients (e.g., iron, zinc, selenium, vitamin C, vitamin A, α-tocopherol, vitamin B12, and folic acid) and essential minerals [60,61,62,63,64,65,66]. It is therefore advisable to follow the changes in the concentration of these molecular determinants in connection with *H. pylori* infection. Fourier transform infrared spectroscopy in conjunction with artificial neural networks enable the analysis of molecular parameters in various biological materials, including blood serum. Taking this into account, the purpose of this study was to use this specialized tool to determine the specific motives of IR spectra of serum samples from *H. pylori*-infected children (*n* = 41), including those with growth disorders vs. uninfected children (*n* = 43). Infrared spectra were measured using an FTIR/FT-NIR Spectrum 400 spectrometer. The IR spectrum was measured in the wavenumber range of 3000–750 cm^−1^ and then subjected to mathematical pre-processing (calculation of first derivative).

For sera obtained from *H. pylori*-positive donors, lower absorbance values were observed for individual molecules than when using sera from *H. pylori*-negative donors. These differences concerned the following components: vitamin A, vitamin B6, vitamin B12, vitamin C, α-tocopherol, folic acid, β-carotene, and lutein as well as the hormone peptides ghrelin and leptin. Lower levels of these molecules in the blood, which were estimated by biochemical or serological methods, were associated with *H. pylori* infection [59,60,61,62,63,64,65,66]. The low levels of vitamin B12 in *H. pylori*-infected patients were linked with an impairment of vitamin B12 absorption from food and the development of pernicious anemia [61,62,67]. The lower concentration of β-carotene (pro-vitamin A) was shown in patients with gastric atrophy and intestinal metaplasia due to *H. pylori* infection [59,63]. Similarly, vitamin C concentration in plasma was 20% lower in *H. pylori*-infected subjects than it was in negative controls, even after correction for confounding factors such as smoking and dietary behavior. *H. pylori* infection may cause an irreversible inactivation of ingested vitamin C in the intestinal lumen prior to its absorption [68,69].

Furthermore, *H. pylori* infection is related to a lower secretion of appetite-regulating peptides and hormones, including ghrelin, which is involved in the hypothalamic regulation of metabolism and energy balance, as well as leptin and gastrin [70,71,72,73,74,75].

Concerning the clinical implications of this study, it seems that lowering all the molecular markers shown using the FTIR technique in conjunction with artificial neural networks may have a negative impact on the health and development of children; it may, however, be helpful in detecting growth delay driven by *H. pylori* infection in children. This combined analysis may also help to predict, on the basis of soluble systemic markers, the potential deleterious effects of *H. pylori* infection on the level of gastric barrier. Further research is needed to standardize this technique for the proposed application. It is expected that it will be possible to propose additional molecular markers to differentiate *H. pylori*-infected patients from uninfected ones in terms of the consequences of this infection.

## 5. Conclusions

Based on the chi-square test, 10 wavenumbers of IR spectra correlating with *H. pylori* infection were selected for ANN design. The sensitivity and specificity of this model were 95% and 86%, respectively, whereas accuracy reached 90%. This study shows that the combination of infrared spectroscopy and ANN techniques may be useful for differentiating serum samples from children exposed or unexposed to *H. pylori*. Particularly, components selected for analysis including vitamin A, vitamin B6, vitamin B12, vitamin C, α-tocopherol, folic acid, β-carotene, and lutein, as well as the hormone peptides ghrelin and leptin can help to diagnose children with growth delay due to *H. pylori* infection. Further study on the larger number of samples will make it possible to standardize this method for investigating molecular level changes correlated with *H. pylori* infection in children and presumable consequent delayed growth. In future research the relationship between the IR parameters determined in this study with the biomarkers of local and systemic inflammatory response in *H. pylori*-infected children should also be considered. Introduction of these additional parameters will enable a more comprehensive analysis of the health status of *H. pylori*-infected patients and could possibly enable the application of specific treatment.

## Figures and Tables

**Table 1:** Molecules identified in the composition IR spectra of children’s sera.

Molecule	Absorption Band (cm^−1^)	ID	Description	Component Group	Reference
α-tocopherol	1472 1078	(B1)	CH_3_/CH_2_ asymmetrical scissoring C-O symmetric stretching of glucose region	Cyclopropane	[42]
β-carotene	1650 1457 1384 1324 1096	(B2)	C=O symmetric stretching asymmetric C-H scissoring of -CH_3_ CH_3_ deformation O-H bending C-C-C bending	Amide I Amino acid	[43]
Folic acid	3050 1485–1519 1604–1619 1650 1693	(B3)	N-H asymmetric stretching CO_2_ asymmetric stretching NH_2_ scissoring NH_2_ scissoring NH_2_ scissoring C=O symmetric stretching	Amino acid Amide I Amide I Amide I Amide I	[44]
Ghrelin	1640	(B4)	NH_2_ scissoring	Amides I	[45]
Leptin	1740 1550	(B5)	C=O stretch N-H in plane bending vibration strongly coupled to C-N stretching vibration protein	Amide II	[46]
Lycopene	3100 2851 1640 1450–1400 1375	(B6)	N-H asymmetric stretching C-H symmetric stretching of CH_2_ group NH_2_ scissoring CH_3_ asymmetric deformation CH_2_ wagging	Amino acid Lipids Amide I	[47]
Lutein	1517–1500	(B7)	CO_2_ asymmetric stretching		[43]
Vitamin B6	1280–1315	(B8)	C-H/N-H deformation vibration models methyl groups	Amide III	[48]
Vitamin B12	3120 1670–1665	(B9)	CH_3_ asymmetric stretching of CH_3_ group C=O bands	Fatty acid Amide I	[49]
Vitamin C	1760 1634 1322	(B10)	C=O stretch NH_2_ scissoring C-H/N-H deformation vibration models methyl groups	Amide I Amide III	[50]

**Table 2:** Best predictors for *H. pylori* infection.

Window	Absorption Band (cm^−1^)	χ^2^ Test Value	*p*-Value (×10^−5^)	One of Possible Chemical Bonds
W4	1060	15.72	7.3	N-H bending
	1087	14.33	15.4	C-N stretch
	1124	17.15	3.4	C-N stretch
	1138	13.03	30.6	C-O stretch
	1139	13.03	30.6	C-O stretch
	1180	13.36	25.7	C-O stretch
W3	1484	12.01	52.9	asymmetric C-H scissoring of -CH_3_
	1493	13.36	25.7	CO_2_ asymmetric stretching
	1495	12.01	52.9	CO_2_ asymmetric stretching
W2	1557	13.76	20.8	N-H bending

**Table 3:** Multilayer perceptrons designed for the detection of *H. pylori* infection.

No.	Topology	Correct Classifications Percentage		Error Function	Activation Function	
		Training Subset	Validation Subset		Hidden Neurons	Output Neurons
1	10-3-2	100%	90%	SOS	Exponential	Linear
2	8-5-2	95%	90%	SOS	Logistic	Exponential
3	9-3-2	100%	88%	SOS	Tanh	Linear
4	9-4-2	100%	88%	SOS	Tanh	Linear
5	10-4-2	100%	86%	Entropy	Tanh	Softmax
6	6-1-2	69%	86%	Entropy	Tanh	Softmax
7	8-3-2	100%	86%	SOS	Logistic	Tanh
8	5-3-2	98%	83%	Entropy	Exponential	Softmax
9	5-2-2	98%	83%	SOS	Tanh	Tanh
10	5-1-2	83%	83%	Entropy	Tanh	Softmax
11	7-1-2	69%	83%	Entropy	Logistic	Softmax
12	7-2-2	95%	83%	Entropy	Logistic	Softmax

SOS: sum of squares; Tanh: hyperbolic tangent.

**Table 4:** Indicators of effectiveness of the 10-3-2 artificial neuron network (ANN).

	Type of Measured Indicator	Value
1	Sensitivity	0.95
2	Miss rate	0.05
3	Specificity	0.86
4	False positive rate	0.14
5	Precision	0.87
6	False discovery rate	0.13
7	False omission rate	0.05
8	Negative predictive value	0.95
9	Positive likelihood ratio	6.67
10	Negative likelihood ratio	0.06
11	Accuracy	0.90
12	Informedness	0.81
13	Markedness	0.82
